# Erythème péri anal douloureux

**DOI:** 10.11604/pamj.2014.18.292.5021

**Published:** 2014-08-14

**Authors:** Fatima Zahra Elfatoiki, Fouzia Hali

**Affiliations:** 1 Service de Dermatologie, CHU Ibn Rochd, Casablanca, Maroc

**Keywords:** Erythème, anite, streptocoque béta-hémolytique, Erythema, anusitis, beta-hemolytic streptococcus

## Image en medicine

Enfant de 3 ans adressé en Dermatologie pour le diagnostic d'un érythème périanal douloureux. Les lésions évoluaient depuis dix jours et n'avaient pas été améliorées après application d'un antimycosique local avec de l’éosine. Cette symptomatologie était apparue dans un contexte de pharyngite fébrile traitée symptomatiquement. L'examen clinique retrouvait un érythème rouge sensible à la palpation bien limité avec une fine desquamation périphérique. Par ailleurs l'enfant était apyrétique, en bon état général. L'examen de la cavité buccale était sans particularité. Le prélèvement bactériologique périanal isolait un streptocoque béta-hémolytique du groupe A. Un traitement par Ampicilline à la dose de 50mg/kg/j pendant 10 jours était instauré avec une disparition des lésions après 48 heures.

**Figure 1:**
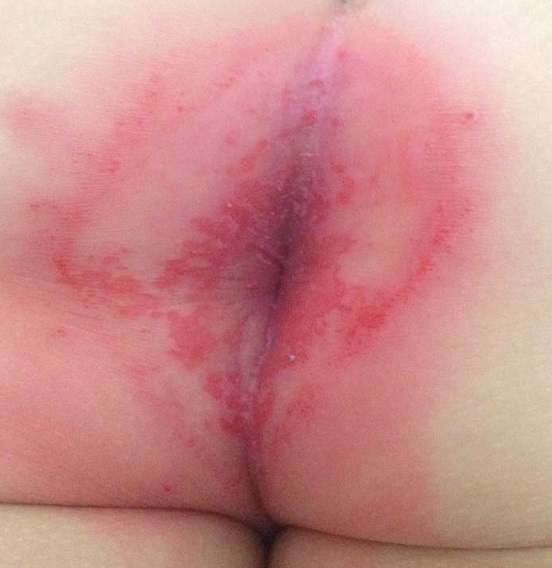
Anite

